# Natural Fish Oil Versus Purified Fish Oil: Composition, Efficacy, and Applications

**DOI:** 10.1155/jnme/5029849

**Published:** 2026-10-15

**Authors:** Jingxu Nie, Linlin Fan, Juan Du, Weihua Wang, Jun Li, Lianzhen Chen

**Affiliations:** ^1^ Department of Pharmacy Shanxi Province Cancer Hospital Shanxi Hospital Affiliated to Cancer Hospital Cancer Hospital Affiliated to Shanxi Medical University Chinese Academy of Medical Sciences Taiyuan Shanxi China cacms.ac.cn; ^2^ Department of Pharmacy National Cancer Center National Clinical Research Center for Cancer Cancer Hospital Peking Union Medical College Chinese Academy of Medical Sciences Beijing China cacms.ac.cn

**Keywords:** biological availability, cardiovascular diseases, fat emulsions, fish oil, isolation and purification

## Abstract

This review proposes a purity–bioavailability–indication framework to systematically differentiate natural fish oil (NFO) and purified fish oil (PFO) and guide clinical decision‐making. We categorize fish oil formulations along a therapeutic gradient from nutritional maintenance to targeted intervention, with molecular structure stratification based on bioavailability and safety profiles. NFO retains natural triglyceride structures with superior absorption kinetics but lower ω‐3 polyunsaturated fatty acid (PUFA) purity, positioning it for maintenance nutritional supplementation. PFO, through advanced purification, achieves higher purity in ethyl esters (EEs), free fatty acids (FFAs), and re‐esterified triglycerides (rTG) forms. The rTG structure uniquely combines high purity with enhanced bioavailability, bridging the gap between NFO and conventional PFO. Within the PFO category, our framework distinguishes eicosapentaenoic acid ethyl ester (EPA‐EE) as the evidence‐based choice for atherosclerotic cardiovascular disease (ASCVD) risk reduction, combinations of EPA and docosahexaenoic acid (EPA/DHA) as neutral for cardiovascular outcomes, and rTG as the bioavailable alternative for enteral therapeutic applications. For parenteral nutrition, PFO‐based lipid emulsions provide formulation and operational advantages. In summary, this framework identifies a fundamental trade‐off between natural matrix preservation (NFO) and purity/bioavailability optimization (PFO). PFO, particularly high‐purity EPA‐EE formulations, demonstrates established efficacy in cardiovascular risk reduction, while rTG offers superior bioavailability with comparable clinical promise; both PFO formulations show advantages in parenteral nutrition. High‐dose ω‐3 formulations, regardless of EPA monotherapy or EPA/DHA combination, carry a dose‐dependent atrial fibrillation risk that warrants age‐stratified assessment. NFO remains preferable for general nutritional supplementation given its favorable tolerability and preservation of natural antioxidants. Selection between NFO and PFO should be guided by clinical indication, required dosage, and arrhythmia risk stratification.

## 1. Introduction

The consumption of fish by humans traces back to the Paleolithic era, with the development of preservation techniques such as smoking and drying facilitating its utilization. Since the 1970s, studies by Bang and Dyerberg et al. on the low incidence of coronary heart disease (CHD) among Greenland Inuit populations drew academic attention to the cardioprotective effects of fish and fish oil [1, 2]. The primary active components of fish oil are ω‐3 long‐chain polyunsaturated fatty acids (PUFAs), which vary in concentration across different fish oil products. These PUFAs predominantly include eicosapentaenoic acid (EPA; 20:5 n‐3), docosahexaenoic acid (DHA; 22:6 n‐3), and minor amounts of docosapentaenoic acid (DPA; 22:5 n‐3) [3]. However, subsequent research challenged these initial findings, revealing no significant difference in coronary artery disease incidence between Inuit and non‐Inuit populations [4]. Despite ongoing controversy, fish oil has gained widespread recognition for its numerous health benefits.

At the molecular level, the beneficial effects of fish oil are attributable to its ability to modulate various signaling pathways, including the nuclear factor kappa‐B (NF‐κB), Toll‐like receptor (TLR), transforming growth factor‐beta (TGF‐β), and peroxisome proliferator‐activated receptor (PPAR) pathways [5]. Multiple studies demonstrate that ω‐3 PUFAs play significant roles in promoting cardiovascular health, reducing inflammation, regulating immune function, and enhancing tumor radiosensitivity [6–9]. They exert favorable regulatory effects on diverse health biomarkers, including lowering plasma cholesterol and triglyceride levels, improving fat tolerance, and reducing platelet adhesiveness [10]. Regular ω‐3 PUFA intake is associated with improved brain development, enhanced learning and memory, and better visual function [6]. Additionally, fish oil supplementation has shown promising results in alleviating symptoms of autoimmune and inflammatory disorders, such as rheumatoid arthritis (RA) and inflammatory bowel disease [8, 11]. These findings continue to drive research into the mechanistic actions and potential clinical applications of ω‐3 PUFAs.

Natural fish oil (NFO), also termed triglyceride form fish oil, represents the natural form of ω‐3 in fish. Its ω‐3 PUFAs purity is typically low; “purity” refers to the proportion of ω‐3 PUFAs (such as DHA and EPA) in the final product (all components). [Note: Concentration denotes the absolute content of ω‐3 PUFAs (DHA and EPA) per unit of fish oil] [12]. NFO requires higher intake doses to achieve therapeutic effects but exhibits relatively higher absorption rates [13–16]. To address this limitation, researchers have developed various purification technologies to produce purified fish oil (PFO) with substantially enhanced purity, now exceeding 90% ω‐3 PUFAs content [15, 17]. The absorption and utilization efficacy of ω‐3 PUFAs varies across products of different purities and structural forms.

Despite extensive research on ω‐3 PUFAs, existing literature lacks a unified framework to guide clinical selection between NFO and PFO, with most reviews treating them as interchangeable rather than distinct therapeutic categories. The relationship between molecular structure and clinical outcomes remains unresolved. Conflicting trial results, with EPA ethyl ester (EPA‐EE) demonstrating benefit in REDUCE‐IT but EPA/DHA combinations showing neutral effects in STRENGTH and OMEMI, have generated uncertainty regarding optimal composition and dosing [1, 18, 19]. Safety profiles are inadequately stratified by formulation. Direct comparisons between NFO and PFO in parenteral nutrition are also absent. This review systematically evaluates these gaps and proposes a purity–bioavailability–indication framework to resolve these uncertainties and guide clinical selection.

## 2. Literature Search Strategy

This narrative review was conducted by systematically searching PubMed/MEDLINE, Embase, and the Cochrane Library for English‐language literature published up to July 2026. Search terms included “omega‐3 fatty acids,” “fish oil,” “eicosapentaenoic acid,” “docosahexaenoic acid,” “ethyl ester,” “re‐esterified triglyceride,” “cardiovascular disease,” and “parenteral nutrition.” We prioritized high‐quality evidence from large‐scale randomized controlled trials and meta‐analyses, followed by pharmacokinetic studies and clinical guidelines. Trial selection was based on relevance to the purity–bioavailability–indication framework, sample size, and methodological rigor. This is a narrative review; therefore, data were synthesized thematically rather than through systematic quantitative pooling.

### 2.1. Preparation Process of NFO and PFO

#### 2.1.1. NFO

According to the European Pharmacopoeia 11.0 definition, NFO is rich in ω‐3 fatty acids that were purified, winterized, deodorized, and obtained from fish of families such as Engraulidae, Carangidae, Clupeidae, Osmeridae, Scombridae, and Ammodytidae (Type I NFO), or from the genera Thunnus and Sarda within the family Scombridae (Type II NFO). The ω‐3 fatty acids are defined as the following acids: alpha‐linolenic acid (C18:3 n‐3), moroctic acid (C18:4 n‐3), eicosatetraenoic acid (C20:4 n‐3), timnodonic (eicosapentaenoic) acid (C20:5 n‐3; EPA), heneicosapentaenoic acid (C21:5 n‐3), clupanodonic acid (C22:5 n‐3), and cervonic (docosahexaenoic) acid (C22:6 n‐3; DHA). Fish oil composition is highly complex, varying primarily according to the source species [20]. EPA and DHA constitute the main ω‐3 PUFAs in NFO, with total ω‐3 PUFAs content in triglyceride form exceeding 28% in both Type I and Type II NFO to ensure efficacy. These essential fatty acids originate from algae and phytoplankton, accumulating in fish through marine trophic transfer [20]. NFO also contains abundant antioxidants (e.g., iodine, selenium, and vitamin E) that protect oxidation‐prone lipids [21]. Vitamin E concentrations typically range from 600 to 7000 μg/g in most products [22, 23]. Additional components include sterols, squalene, vitamin A, and vitamin D. NFO is typically extracted from fish subcutaneous fat while preserving the natural triglyceride structure, enhancing human absorption. Extraction methods include using biosolvents such as d‐limonene, effectively recovering ω‐3 PUFAs and natural antioxidants like lutein [24].

In its natural state, fish oil appears as a pale‐yellow liquid and may contain various contaminants, including organochlorine pesticides, polychlorinated biphenyls (PCBs), heavy metals, and polycyclic aromatic hydrocarbons (PAHs) [22, 25]. To address this issue, multiple methods have been developed to remove contaminants while preserving the nutritional quality of fish oil. Techniques such as activated carbon adsorption, deodorization, silica gel column chromatography, enzymatic treatment, and supercritical CO_2_ extraction have demonstrated varying degrees of effectiveness in eliminating different pollutants and enhancing EPA/DHA purity. Combining these methods can significantly reduce contaminant levels to comply with production standards [26, 27]. Nevertheless, NFO intended for pharmaceutical, food industry, and dietary supplement applications may still contain residual contaminants [28]. Therefore, regular monitoring of both traditional and emerging contaminants in fish oil products is essential to ensure patient safety.

Higher purity correlates with reduced impurities (e.g., saturated fats, cholesterol), improved safety, and greater suitability for specific health needs (e.g., lipid‐lowering). International guidelines recommend selecting fish oil with ≥ 80% purity for targeted health requirements. Adults require a daily intake of at least 250–500 mg of ω‐3 PUFAs to meet essential health needs.

#### 2.1.2. PFO

According to the definition in the European Pharmacopoeia 11.0, PFO is a mixture of monoesters, diesters, and triesters formed by esterification of ω‐3 fatty acids with glycerol, mainly in the form of triglycerides. It can be obtained either by esterification of concentrated and purified ω‐3 fatty acids with glycerol or by transesterification of ω‐3 fatty acid EEs with glycerol. The total content of ω‐3 fatty acids in triglyceride form in this mixture must be at least 60.0% to be effective. The origin of the ω‐3 acids is the body oil obtained from fish of families such as Engraulidae, Carangidae, Clupeidae, Osmeridae, Salmonidae, and Scombridae, or from animals of the class Cephalopoda. The ω‐3 acids are identified as the following acids: alpha‐linolenic acid (C18:3 n‐3), moroctic acid (C18:4 n‐3), eicosatetraenoic acid (C20:4 n‐3), timnodonic (eicosapentaenoic) acid (C20:5 n‐3; EPA), heneicosapentaenoic acid (C21:5 n‐3), clupanodonic acid (C22:5 n‐3), and cervonic (docosahexaenoic) acid (C22:6 n‐3; DHA). During fish oil purification, it is crucial to remove contaminants that affect quality and shelf life while preserving beneficial fatty acids such as EPA and DHA [29]. The refining process typically includes steps such as degumming, deacidification, bleaching, and deodorization [30]. These steps significantly reduce harmful compounds like dioxins/furans, PCBs, and arsenic while maintaining good fatty acid content [29]. To address the issue of variable components in fish oils from different sources, various refining techniques have been developed, including traditional physical/chemical methods and nontraditional biological and membrane process technologies [31]. Chemical refining can also alter the composition of volatile compounds and increase the proportion of PUFAs [30]. Due to the high susceptibility of fish oil to oxidation, researchers are exploring stabilization techniques using natural antioxidants as alternatives to synthetic antioxidants [32].

With recent advances in fish oil purification technology, manufacturers have developed various high‐purity product forms. Depending on the extraction method and structure, high‐purity ω‐3 PUFA products primarily exist in three structural forms: EEs, free fatty acids (FFAs) (also known as nonesterified fatty acids), and re‐esterified triglycerides (rTG). These are described in detail below.

##### 2.1.2.1. EE

EE‐type PFO is combined in the form of fatty acids and ethanol through chemical esterification and molecular distillation technology (or supercritical extraction/chromatography). In the molecular structure of EE‐type PFO, ω‐3 PUFAs exist as EEs. This structure lacks the primary glycerol backbone and cannot be directly absorbed by human cells. The additional metabolic steps increase the complexity of EE‐type PFO being absorbed by the human body, and thus, its absorption and utilization rate are relatively lower [33]. During human digestion and absorption, EE‐type PFO first needs to be emulsified by bile salts in the small intestine and then hydrolyzed by carboxyl ester lipase. This hydrolysis process releases FFAs and ethanol molecules. The released fatty acids are subsequently absorbed by intestinal cells and, within these cells, reconverted into triglyceride form before they can be absorbed and utilized by the body.

Production of high‐purity EPA‐EE involves extraction, refining, and ethyl esterification processes [13]. To enhance purity and inhibit oxidation, researchers have incorporated tea polyphenol palmitate during urea complexation and molecular distillation while optimizing process parameters, achieving EPA‐EE purity levels of up to 96.95% [34].

##### 2.1.2.2. FFA

Hydrolysis of EE‐type ω‐3 PUFAs into FFA cleaves the EE bond under acidic or alkaline conditions, or via enzymatic catalysis, releasing the free form of ω‐3 PUFAs. The ω‐3 PUFAs in FFA‐type PFO exist as FFAs unbound to glycerol molecules. Posthydrolysis, the resulting FFA‐type ω‐3 PUFAs may be exposed to air, increasing oxidation risk. Antioxidant treatment is therefore essential to protect FFA‐type ω‐3 PUFAs from oxidation. This typically involves adding antioxidants such as vitamin E (tocopherols) and other natural or synthetic antioxidants, which scavenge free radicals to prevent oxidative degradation of ω‐3 PUFAs. Compared to the EE form, FFA‐type PFO is not dependent on hydrolysis enzyme activity and therefore exhibits higher bioavailability, particularly under low‐fat dietary conditions. Studies indicate that the bioavailability of FFA‐type PFO is four times higher than that of the EE form [35].

##### 2.1.2.3. rTG

rTG‐type PFO innovatively combines the advantages of NFO and EE by converting EE‐type ω‐3 PUFAs back into a triglyceride form resembling NFO through re‐esterification, achieving dual benefits of high purity and high absorption. Taking the mean increase in EPA plus DHA in all three serum lipid fractions (cholesterol esters, triglycerides, and phospholipids) for NFO as unity (100%), the mean relative bioavailability of EPA plus DHA from rTG‐type PFO (124%) outperforms both NFO (100%) and EE (73%) [36]. The degree of re‐esterification plays a crucial role in rTG formulations, significantly influencing their performance in lipidomic pharmacodynamics. Specifically, higher rTG percentages in rTG‐type PFO correlate with increased EPA and DHA content in erythrocyte and serum phospholipids. This suggests that the rTG percentage may serve as a key chemical characteristic/quality control metric, impacting the in vivo bioavailability and biomembrane integration of rTG‐type PFO [15]. These findings demonstrate that the structural form of ω‐3 PUFA supplements is a critical determinant of bioavailability [37].

### 2.2. Fatty Acid Composition and Bioavailability Comparison

The compositional profile of ω‐3 PUFAs varies depending on the purification methods. PFO exhibits a higher content of total ω‐3 PUFAs in the form of triglycerides, as well as content of EPA and DHA in the form of triglycerides, compared to NFO. Bioavailability has been shown to be greater with FFA formulations than with EE formulations, as FFA forms are dependent on neither digestive enzymes nor fat for absorption [33]. The lower efficiency of the hydrolysis and absorption of EE is the lower rate of hydrolysis of the ester bond by carboxyl ester lipase and the inefficient provisioning process of glycerol necessary for transport [33]. Bioavailability refers to the extent to which a drug or nutritional supplement is absorbed and utilized after entering the body. NFO, which retains the natural triglyceride structure, demonstrates higher absorption rates, with bioavailability superior to the EE form [38], while rTG‐type PFO bioavailability exceeds that of NFO (Figure 1). In accordance with the European Pharmacopoeia and the literature [34, 36, 39], we compare the fatty acid composition and bioavailability of different NFO and PFO product types in Table 1.

**FIGURE 1:**
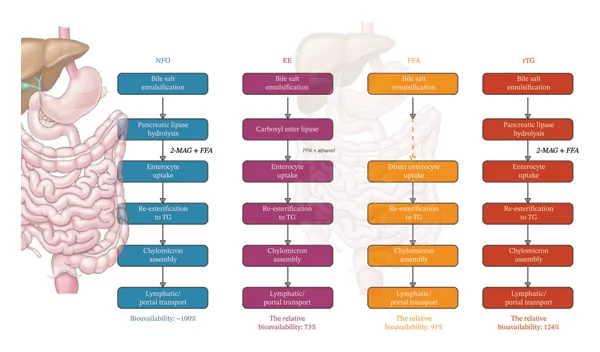
Comparative absorption kinetics of ω‐3 PUFA formulations in the gastrointestinal tract. Note: 2‐MAG, 2‐monoacylglycerol; CM, chylomicron; EE, ethyl ester; FFA, free fatty acid; NFO, natural fish oil; rTG, re‐esterified triglyceride; TG, triglyceride. Solid arrows indicate the normal pathway. Dashed arrows indicate a bypass pathway.

**TABLE 1:** Purity and bioavailability requirements for different fish oil product types.

	EPA/total fatty acids (%)	DHA/total fatty acids (%)	Total ω‐3 fatty acids/total fatty acids (%)	Bioavailability of EPA/DHA (%)
Natural fish oil (NFO)				
Type I NFO	≥ 13.0	≥ 9	≥ 28	≈100
Type II NFO	4.0∼12.0	≥ 20	≥ 28	≈100
Purified fish oil (PFO)				
Re‐esterified triglycerides (rTG)	28.5	19.8	≥ 58∼96.95	124
Ethyl ester (EE)	28.8∼96.95	21.4∼37.5	≥ 90∼96.95	73
Free fatty acids (FFAs)	33.5	21.5	≥ 58∼96.95	91

*Note:* EPA: eicosapentaenoic acid; DHA: docosahexaenoic acid; total ω‐3 fatty acids: EPA + DHA.

### 2.3. Process Balance Between Nutrient Retention and Contaminant Removal

Fish oil contains beneficial PUFAs such as EPA and DHA, along with other micronutrients, including vitamins, cholesterol, and phospholipids [32]. However, refining processes for contaminant removal may eliminate natural antioxidants (e.g., lipophilic polyphenols), negatively impacting oil stability [40, 41]. Among various refining methods, activated carbon treatment and deodorization primarily remove pigments, odors, and harmful substances (e.g., polychlorinated dibenzo‐p‐dioxins and dibenzofurans [PCDD/Fs] and PCBs) via adsorption, with relatively minor effects on nutritional components [26, 29]. Short‐path distillation effectively eliminates persistent organic pollutants (POPs), achieving reduction rates of 76%–96% for PCDD/Fs, 89%–99% for PCBs, and > 86% for polybrominated diphenyl ethers (PBDEs). However, its processing conditions may cause volatile nutrient losses, including cholesterol and vitamins A, D, and E [42, 43]. Higher temperatures and lower pressures enhance contaminant removal efficiency but typically exacerbate nutrient degradation [43]. However, although there is a loss of vitamin content, short‐path distillation can still maintain a sufficient PUFA level, but it may reduce the oxidation stability of fish oil because the removal of natural antioxidants and high‐temperature and low‐pressure conditions will promote the oxidation of fish oil. Modeling optimal processing parameters is recommended to balance contaminant removal and nutrient retention—e.g., achieving > 76% reduction in WHO‐PCDD/F‐PCB‐TEQ while limiting vitamin loss to < 20%—to simultaneously meet oil quality and regulatory safety standards [42].

### 2.4. Food, Therapeutic Applications, and Safety

#### 2.4.1. Applications in Food for Special Medical Purposes (FSMPs)

Fish oil is increasingly used in nutraceuticals [44], yet its high oxidative sensitivity and unpleasant fishy odor pose challenges for FSMP applications. Current technologies to enhance oxidative stability and mask odors include direct antioxidant addition, emulsification, and microencapsulation [45]. Compared to NFO, purification methods for PFO eliminate fishy odors, improving patient acceptance. The food industry has successfully incorporated fish oil into diverse products, including infant formula, dairy products, and meat/poultry items [46]. Manufacturers are developing eco‐friendlier extraction and purification technologies to maximize yield while minimizing nutrient degradation [32]. Despite public concerns about seafood and fish oil product safety, rigorously tested fish, shellfish, and high‐concentration PFO products rarely pose safety issues [47, 48]. Existing literature confirms that both NFO and PFO are generally safe for food industry use [32].

#### 2.4.2. Clinical Applications

##### 2.4.2.1. Cardiovascular Disease Management

###### 2.4.2.1.1. Dyslipidemia and Atherosclerotic Cardiovascular Disease (ASCVD) Prevention

NFO and PFO demonstrate divergent cardiovascular efficacy depending on fatty acid composition and molecular structure. PFO formulations have been extensively evaluated in large‐scale cardiovascular outcome trials (Table 2), with high‐dose EPA‐EE (icosapent ethyl, 4 g/day) reducing major cardiovascular events by 25% in patients with ASCVD or diabetes and elevated triglycerides (135–499 mg/dL) in REDUCE‐IT and showing similar benefit in RESPECT‐EPA, independent of triglyceride lowering [1, 50]. The OCEAN study revealed that EE‐type PFO (90% ω‐3 PUFAs) significantly lowered serum triglycerides and altered serum lipidomic profiles in patients with Type 2 diabetes and hypertriglyceridemia compared with corn oil. Conversely, EPA/DHA combinations in STRENGTH and OMEMI failed to demonstrate cardiovascular protection despite comparable triglyceride reductions, suggesting DHA may attenuate EPA‐mediated benefits or that formulation characteristics influence outcomes beyond lipid effects [18, 19]. Lower‐dose EPA/DHA EE (VITAL, ASCEND) showed modest reductions in fatal myocardial infarction and cardiovascular death without primary endpoint benefit [49, 51].

**TABLE 2:** Summary of PFO cardiovascular endpoint event studies.

Trial (year published)	Inclusion criteria	ω‐3 PUFAs formulation	Therapeutic dose	Primary endpoint	Primary endpoint HR (95% CI), *p* value
ASCEND (2018) [49]	Patients ≥ 40 years w/DM but no CVD evidence	EPA + DHA EE	1 g/day	Nonfatal MI, nonfatal stroke, TIA, vascular death	0.97 (0.87–1.08) *p* = 0.55

VITAL (2019) [50]	Men ≥ 50 years and women ≥ 55 years, no CVD evidence	EPA + DHA EE	1 g/day	(1) MI, stroke, or CV death and (2) invasive cancer	(1) 0.92 (0.8–1.06) *P* = 0.24 (2) 1.03 (0.93–1.13) *p* = 0.56

REDUCE‐IT (2019) [1]	TG levels 150–499 mg/dL w/ASCVD or diabetes w/≥ 1 other risk factors	EPA‐EE	4 g/day	CV death, nonfatal MI, nonfatal stroke, coronary revasc, or unstable angina	0.75 (0.68–0.83) *p* < 0.001

STRENGTH (2020) [18]	TG levels 180–499 mg/dL and HDL‐C ≤ 40 mg/dL w/ASCVD or diabetes w/≥ 1 other risk factors	EPA + DHA FFA	4 g/day	CV death, nonfatal MI, nonfatal stroke, coronary revasc, or unstable angina	0.99 (0.90–1.09) *p* = 0.84

OMEMI (2021) [19]	Elderly patients (70–82 years) w/recent AMI	EPA + DHA FFA	1.8 g/day	AMI, revasc, stroke, all‐cause death, or HF hospitalization	1.08 (0.82–1.41) *p* = 0.60

RESPECT‐EPA (2022) [48]	EPA/AA ratio < 0.4 Chronic CAD ≥ 1‐month prior statin use	EPA‐EE	1.8 g/day	CV death, nonfatal MI, nonfatal stroke, revasc, unstable angina	0.79 (0.62–1.00) *p* = 0.055

*Note:* ASCVD, atherosclerotic cardiovascular disease; DHA, docosahexaenoic acid; EPA, eicosapentaenoic acid; PUFA, polyunsaturated fatty acids; revasc, revascularization; TG, triglyceride.

Abbreviations: AA, arachidonic acid; AMI, acute myocardial infarction; CAD, coronary artery disease; CI, confidence interval; DM, diabetes mellitus; EE, ethyl ester; EOS, end‐of‐study; FFA, free fatty acid; HDL‐C, high‐density lipoprotein cholesterol; HF, heart failure; HR, hazard ratio; ICD, implanted cardioverter/defibrillator; LDL‐C, low‐density lipoprotein cholesterol; MI, myocardial infarction; TIA, transient ischemic attack.

Regarding molecular structure, rTG exhibits superior bioavailability (124%) and tissue incorporation compared with EE (73%), though no large‐scale cardiovascular outcome trials have evaluated rTG formulations [36]. Clinical trials such as EVOLVE demonstrated that FFA‐type PFO—whether as monotherapy or combined with statins—effectively reduced triglycerides and non‐HDL cholesterol in patients with severe hypertriglyceridemia [52, 53]. A dose of 4 g/day provided greater benefits than 2 g/day. FFA formulations were generally well‐tolerated, with gastrointestinal side effects being the most commonly reported. These findings suggest that FFA may offer therapeutic advantages over EE in managing hypertriglyceridemia, particularly in patients on low‐fat diets.

NFO retains favorable absorption kinetics but lacks modern cardiovascular outcome evidence at therapeutic doses, limiting its role in prescription cardiovascular risk modification.

###### 2.4.2.1.2. Heart Failure

ω‐3 PUFAs provide modest but significant benefits in chronic heart failure (HF). The GISSI‐HF trial randomized 6975 patients with New York Heart Association (NYHA) Class II–IV HF to ω‐3 PUFAs 1 g daily (predominantly EPA/DHA EE) or placebo for 3.9 years, demonstrating reduced all‐cause mortality (27% versus 29%, adjusted HR 0.91) and the composite of death or CV hospitalization (57% versus 59%, adjusted HR 0.92) [54]. No direct comparisons between NFO and PFO formulations exist in HF populations. The trial employed a low‐dose EE preparation, and whether higher dose purified EPA or alternative molecular structures (rTG, FFA) confer additional benefit remains uncertain. NFO retains theoretical absorption advantages but lacks HF outcome data.

##### 2.4.2.2. Inflammatory and Autoimmune Conditions

ω‐3 PUFAs demonstrate anti‐inflammatory effects in RA, though no studies directly compare NFO and PFO formulations within autoimmune populations. Clinical trials in RA report reduced disease activity and nonsteroidal anti‐inflammatory drug (NSAID) requirements with fish oil supplementation, but formulation types are unspecified [55, 56]. Inconsistent results in inflammatory bowel disease and psoriasis suggest variable responsiveness, with no evidence favoring specific molecular structures [55].

##### 2.4.2.3. Neurological and Psychiatric Applications

ω‐3 PUFAs demonstrate antidepressant efficacy in major depressive disorder (MDD) and cognitive function; however, no studies directly compare NFO and PFO formulations in psychiatric or neurological populations. Meta‐analysis indicates that supplements with EPA ≥ 60% of total EPA/DHA show significant benefit (effect size 0.532, 95% CI 0.277–0.733, *p* < 0.001), whereas formulations with EPA < 60% are ineffective. Efficacy appears determined by EPA dose in excess of DHA within the range of 200 to 2200 mg daily, suggesting that high‐purity EPA formulations may offer advantages over mixed EPA/DHA compositions [57]. Whether molecular structure or fatty acid composition influences neuropsychiatric efficacy requires direct comparative evaluation.

##### 2.4.2.4. Cancer Supportive Care

ω‐3 PUFAs demonstrate supportive benefits in cancer cachexia; however, no studies directly compare NFO and PFO formulations in oncology populations. Clinical trials report improved weight maintenance and quality of life with EPA and DHA supplementation, but formulation types are unspecified, and no optimal EPA/DHA composition has been established [58]. Preclinical evidence suggests FFA EPA may be effective in cachexia, where EE forms are ineffective at equivalent doses; yet, this has not been translated to clinical evaluation [59]. Evidence for chemotherapy‐induced peripheral neuropathy prevention and radiosensitization remains inconclusive [60]. Whether molecular structure influences efficacy in cancer supportive care requires direct comparative trials.

##### 2.4.2.5. Parenteral Nutrition

Beyond oral fish oil products, fish oil–containing intravenous lipid emulsions (FOC‐IVLEs) have gained widespread use in parenteral nutrition. Importantly, because intravenous administration bypasses the gastrointestinal tract, the oral bioavailability hierarchy shown in Figure 1 does not apply in this setting; instead, PFO‐based emulsions offer advantages in purity and formulation standardization. FOC‐IVLEs derive from diverse sources, including NFO, PFO, blended fish oils, and synthetic fish oils—predominantly sourced from NFO through refining and purification processes. Fish oil–containing lipid emulsions are recognized as fourth‐generation formulations due to their significant compositional, functional, and clinical advancements. Based on processing methods, they are categorized as NFO or PFO types. According to European Pharmacopoeia standards, PFO requires higher EPA/DHA content than NFO. Clinically used NFO (e.g., ω‐3 fish oil fat emulsion injection) and PFO formulations [e.g., ω‐3‐fish oil medium‐ and long‐chain fat emulsion injection; ω‐3 acid triglycerides (2%) medium‐ and long‐chain fat emulsion/amino acids (16)/glucose (36%) injection; ω‐3 acid triglycerides (2%) medium‐ and long‐chain fat emulsion/amino acids (16)/glucose (16%) injection] all meet pharmacopeial specifications and clinical requirements.

###### 2.4.2.5.1. NFO‐Based Lipid Emulsions

Multiple randomized controlled trials indicate that NFO lipid emulsions (containing 10% fish oil) may reduce mortality and mechanical ventilation duration in critically ill ICU patients through anti‐inflammatory and immunomodulatory effects. However, they show no significant impact on infection rates or ICU length of stay [61]. Insufficient clinical data preclude routine recommendation of NFO emulsions in critical care settings, warranting larger, rigorously designed trials to evaluate their efficacy. Notably, NFO emulsions should generally be administered concurrently with other lipid emulsions. Exceptions include documented soybean oil allergy or pediatric parenteral nutrition–associated cholestasis (PNAC), where short‐term monotherapy with NFO emulsions may be considered [62].

###### 2.4.2.5.2. PFO‐Based Lipid Emulsions

A prospective randomized controlled multicenter trial by Stanislaw et al. demonstrated that after 8 weeks of home parenteral nutrition, ω‐3 PUFAs‐enriched PFO emulsions—compared with traditional standard fat emulsions—induced favorable alterations of fatty acid profiles in plasma and erythrocytes due to enhanced ω‐3 PUFA delivery. PFO emulsions exhibited excellent safety and tolerability, particularly regarding hepatic impact [63]. For patients undergoing orthotopic liver transplantation due to end‐stage liver disease or hepatocellular carcinoma, PFO emulsion‐based parenteral nutrition significantly improved hepatic injury, reduced infectious complications, and shortened postoperative hospitalization [64].

###### 2.4.2.5.3. PFO‐Based Multichamber Bag (MCB) Formulations: Operational Advantages Over Manual NFO Supplementation

Lipid emulsions require coinfusion with amino acids, glucose, vitamins, and trace elements. International guidelines recommend “all‐in‐one” (AIO) infusion. This infusion method is more in line with human physiology and conducive to nutrient metabolism compared with tandem and single‐bottle infusion. Intravenous fish oil emulsions also need to be added to the AIO bag for coinfusion [65]. The commonly used industrialized MCB in clinical practice, including three‐chamber bags and double‐chamber bags, provides a standardized and convenient administration method for these lipid emulsions. This method ensures accurate nutritional ratios and timely delivery while also reducing errors in the formulation of complex parenteral nutrition solutions, as well as reducing errors and bloodstream infections by minimizing complicated preparation steps [66, 67]. Within our purity–bioavailability–indication framework, MCB formulations specifically embody the PFO advantage in parenteral nutrition. NFO emulsions require manual extraction from separate bottles and addition to three‐chamber bags, whereas PFO‐based MCB integrates ω‐3 PUFAs as a standardized, presealed component, eliminating compounding variability and contamination risk. From the perspective of clinical convenience, compared with the single‐bottle NFO fish oil + three‐chamber bags of lipid/amino acid/glucose, the PFO infusion method of the ω‐3 acid triglycerides medium‐ and long‐chain fat emulsion/amino acids/glucose triple‐chamber bag eliminates the operation of extracting fish oil from the bottle and adding it to the three‐chamber bag, which is convenient to use and reduces contamination of the nutrient solution. This operational distinction reinforces the framework’s recommendation that PFO, delivered via MCB, is the preferred formulation for parenteral nutrition applications, whereas NFO remains reserved for scenarios requiring specific natural matrix preservation, such as pediatric PNAC or documented soybean oil allergy.

#### 2.4.3. Adverse Reactions and Safety Considerations

##### 2.4.3.1. NFO Safety Profile

Studies in healthy populations using NFO have reported no increased risks of bleeding, stroke, or mortality, nor elevated incidence of gastrointestinal discomfort or other minor adverse effects (e.g., pruritus, musculoskeletal pain) [68]. NFO retains natural antioxidants that may enhance oxidative stability, though contaminant residues remain a theoretical concern despite purification processes.

##### 2.4.3.2. PFO Safety Profile

###### 2.4.3.2.1. General Safety

For PFO oral formulations in elderly patients with established cardiovascular disease or cardiovascular risk, some studies documented major bleeding complications postsurgery. However, the incidence of major bleeding was identical (1.3%) between PFO intervention groups and controls (placebo, no treatment, flaxseed oil, or olive oil) [69, 70]. Gastrointestinal discomfort was reported significantly more frequently in PFO groups versus controls, though overall symptoms were infrequent, affecting approximately 3.5% of PFO‐treated subjects. Minor adverse events like pruritus and musculoskeletal pain were also reported at low frequencies [71]. Thus, PFO ω‐3 PUFAs formulations are generally safe at appropriate dosages, including for elderly and cardiovascular disease patients.

###### 2.4.3.2.2. EE‐Specific Concern: Atrial Fibrillation (AF) Risk

High‐dose EPA‐EE significantly increases AF risk compared with control groups, with dose‐dependent effects also observed in EPA/DHA combinations. In REDUCE‐IT, icosapent ethyl 4 g/day resulted in 5.3% AF incidence versus 3.9% with mineral oil placebo (*p* = 0.003) [1, 60]. Similar patterns emerged in other major trials. STRENGTH (4 g/day EPA/DHA combination, corn oil comparator) showed 2.2% AF incidence versus 1.3% (HR 1.69), and OMEMI (1.8 g/day, corn oil placebo) showed 7.2% AF incidence versus 4.0% (HR 1.84, *p* = 0.06) [18, 19]. Notably, the higher absolute AF incidence in OMEMI compared with REDUCE‐IT reflects the ultra‐elderly composition of the OMEMI cohort (70–82 years), in whom baseline AF risk is substantially elevated due to age‐related atrial remodeling and comorbidity burden. When adjusted for baseline risk, the hazard ratios across trials demonstrate comparable relative risk elevation with both EPA‐EE and EPA/DHA combinations, indicating a dose‐dependent formulation‐class effect rather than superior safety of either composition. The higher absolute rate in ultra‐elderly populations underscores the importance of age‐stratified risk assessment when prescribing high‐dose ω‐3 formulations. A propensity‐matched cohort of 17,635 patients found a 5.32% versus 3.99% cumulative incidence (HR 1.24–1.26) [72]. A meta‐analysis of 8 RCTs (83,112 participants) demonstrated a dose‐dependent AF risk: > 1 g/day showed a 51% increased relative risk versus 12% for ≤ 1 g/day [73]. These findings suggest that high‐dose ω‐3 formulations, regardless of EPA monotherapy or EPA/DHA combination, carry a dose‐dependent AF risk that warrants caution in patients with preexisting arrhythmia risk factors, particularly in ultra‐elderly populations.

###### 2.4.3.2.3. Comparative Safety Across PFO Formulations

No direct head‐to‐head safety comparisons exist among EE, FFA, and rTG formulations. A network meta‐analysis examined cardiovascular efficacy across multiple formulations, including EE, TG, and rTG, but safety outcomes were not stratified by molecular structure [74]. Indirect evidence suggests prescription EPA‐EE carries a higher bleeding risk compared with other ω‐3 types, and ω‐3 supplementation broadly increases AF risk (RR 1.25) [75]. However, meta‐analyses on bleeding and AF examine ω‐3 PUFAs as a class without distinguishing EE, FFA, or rTG structures [76, 77]. The influence of molecular structure on adverse event rates remains unexamined in direct comparative trials, representing a substantial evidence gap for safety differentiation among purified formulations.

## 3. Conclusion

Based on currently available evidence, PFO, particularly EPA‐EE formulations, demonstrates established therapeutic efficacy in cardiovascular disease prevention, while rTG‐type PFO offers superior bioavailability as an emerging alternative; both formulations show advantages in parenteral nutrition applications. NFO remains suitable for general nutritional supplementation and food fortification where high‐dose therapeutic intervention is not required, given its favorable tolerability and preservation of natural antioxidants, though larger doses are needed to achieve comparable ω‐3 PUFAs intake. For patients with established ASCVD or severe hypertriglyceridemia requiring high‐dose intervention, EPA‐EE is the evidence‐based preferred choice, with rTG as a bioavailable alternative, with monitoring for AF risk with high‐dose ω‐3 formulations, particularly in ultra‐elderly populations. This dichotomy reflects a fundamental trade‐off between purity/bioavailability optimization and natural matrix preservation. Age‐stratified risk assessment is essential given the dose‐dependent AF class effect observed across EPA‐EE and EPA/DHA combinations.

## Author Contributions

Jingxu Nie, Linlin Fan, Juan Du, Weihua Wang, Jun Li, and Lianzhen Chen were involved in the conception and design, analysis, and interpretation of the data. Jingxu Nie and Linlin Fan drafted the paper. Juan Du, Weihua Wang, Jun Li, and Lianzhen Chen critically revised it for intellectual content. Jun Li and Lianzhen Chen provided final approval of the version to be published.

## Funding

This study was sponsored by the Chinese Academy of Medical Sciences Innovation Fund for Medical Sciences (2021‐JKCS‐011) and the National Key Research and Development Program of China (2016YFA0201504), and the Qiangfan Program 2026 Special Fund of Beijing Injury Repair Association (QF‐2026‐01‐09).

## Disclosure

All authors agreed to be accountable for all aspects of the work.

## Conflicts of Interest

The authors declare no conflicts of interest.

## Data Availability

The data that support the findings of this study are available from the corresponding authors upon reasonable request.
